# Supplementary material for: Evaluation of economic strengthening in South Africa and its impact on HIV, sexually transmitted infections, and teenage births: A modelling study
Source: PLoS Med. 2025 Dec 18;22(12):e1004826. doi: 10.1371/journal.pmed.1004826 (PMC12714221; doi:10.1371/journal.pmed.1004826)
Supplement: Attachment — Submitted filename: Reviewer responses2.docx [file pmed.1004826.s004.docx]

Dear Dr Farrell

Thank you very much for considering our paper. We are grateful to you and the reviewers for the helpful comments and suggestions, which have helped to improve the paper substantially. We have revised the paper based on the feedback received, and have responded below to the individual comments. Because of the changes to the prior distributions (in response to point c by reviewer 1) and the doubling of the posterior sample size (in response to point f by reviewer 1), the results have changed slightly from those in the first submission, but the main conclusions are unchanged.

We have also responded to the general technical comments at the end of this response letter. Please let us know if there are any further concerns.

Yours sincerely

Leigh Johnson

Comments from the academic editor:

The methods could be a bit better organized to reflect the main output/results. It does seem that two of the main purposes was to evaluate population attributable fractions for various socioeconomic factors and then also the effects of economic empowerment interventions.

**RESPONSE**: There are separate paragraphs in the Methods section describing the PAF analysis and the future intervention scenarios, but perhaps the structure was not clear. To make things clearer, we have removed the previous ‘Model scenarios’ heading, added a ‘Population attributable fractions’ heading, and added a heading before the last paragraph: ‘Future intervention scenarios’.

Please include the proximal, intermediate, and distal impacts of the economic empowerment interventions. Currently the intermediate is missing (e.g., types of partners, condom use, type of sex, etc.).

**RESPONSE**: We have added the intermediate outcomes in Table 4, which is shown below. We have also added the following paragraph to describe the results: “*When considering intermediate behavioural outcomes, the effects of the cash transfer and vocational training interventions are close to zero (Table 4). However, the school support intervention is expected to significantly reduce the proportion of sexually active young females who are not using hormonal contraception, the proportion of adults in concurrent partnerships and the proportion of men engaging in casual sex (the latter two effects being mediated by the effect of education inequitable gender norms, and the effect of inequitable gender norms on male risk behaviour [1]). The school support intervention may also reduce the proportion of adolescents who are sexually experienced and the number of early marriages, although these reductions are not statistically significant (Table 4).*” As noted here, we believe some of the behavioural changes are likely to be mediated by changes in inequitable gender norms, a component of the model that was not previously described. We have therefore added a few rows in Table 1 showing the assumed effect of education on inequitable gender norms, and the assumed effects of inequitable gender norms on sexual behaviour in men.

Table 4: Reduction in behavioural risks and reproductive health outcomes (2025-2040) as a result of different economic strengthening interventions

|  | School  support | Vocational  training | Cash  transfers |
| --- | --- | --- | --- |
| Behavioural outcomes |  |  |  |
| Sexually experienced adolescents | 4% (0 to 10) | 0% (-1 to 1) | 0% (-1 to 1) |
| Sexually active females (15-24) not  using hormonal contraception | **1% (0 to 2)** | 0% (-1 to 1) | 0% (-1 to 1) |
| Married youth (15-24) | 3% (-3 to 9) | 0% (-6 to 4) | 0% (-6 to 4) |
| Condomless sex (ages 15-49) | 0% (0 to 0) | 0% (0 to 0) | 0% (0 to 0) |
| People in concurrent partnerships | **1% (0 to 2)** | 0% (-1 to 1) | 0% (-1 to 1) |
| Client contacts with sex workers | 0% (-2 to 1) | 0% (-1 to 1) | 0% (-1 to 1) |
| Men having casual sex | **2% (1 to 4)** | 0% (-1 to 1) | 0% (-1 to 1) |
| Women having casual sex | 0% (-1 to 2) | 0% (-1 to 1) | 0% (-1 to 1) |
| Reproductive health outcomes |  |  |  |
| New HIV infections (ages 15+) | 1% (-3 to 4) | 0% (-3 to 3) | 0% (-4 to 4) |
| New HIV infections (men) | 1% (-3 to 5) | 0% (-4 to 5) | 0% (-4 to 4) |
| New HIV infections (women) | 1% (-4 to 5) | 0% (-3 to 4) | 0% (-4 to 3) |
| New STIs (ages 15+) | 1% (0 to 2) | 0% (-1 to 1) | 0% (-1 to 1) |
| New STIs (men) | 1% (0 to 2) | 0% (-1 to 1) | 0% (-1 to 1) |
| New STIs (women) | 1% (0 to 2) | 0% (-1 to 1) | 0% (-1 to 1) |
| Births to teenagers | 5% (-1 to 12) | -1% (-15 to 3%) | 0% (-3 to 3) |

95% confidence intervals are shown in brackets, and significant reductions are formatted in bold.

In Figure 3, although the most likely affected metric is shown for each type of intervention, it would be worthwhile to have all 3 on each of the panels. For example, schooling and vocational training could also be expected to affect income.

**RESPONSE**: We have amended Figure 3 as suggested (shown below). We have also modified the description of the results: “The cash transfer intervention would reduce income inequality, with the Palma ratio (the ratio of income in the top decile to that in the four lowest deciles) decreasing from 8.5 to 7.8 by 2030; *school support and vocational training would have more modest effects on income inequality* (Figure 3a). The school support intervention would significantly increase the proportion of South African youth who have completed high school, from 30% to 38% by 2040, *but the other interventions would have no effects on school completion* (Figure 3b). Vocational training interventions would only slightly increase employment levels, *while school support would slightly reduce the fraction of the population employed in the short term, due to young adults remaining in school for longer* (Figure 3c).”

Figure 3: Intervention impacts on socio-economic outcomes

The Palma Ratio (panel A) is defined as the ratio of total income in the top income decile to the total income in the four lowest deciles. In panel (b), the high school completion fraction is the proportion of all 15-24 year old South Africans who have successfully completed grade 12. In panel (c) the denominator is all individuals aged 15-64 (including those in school and those not actively seeking work).

-----------------------------------------------------------

Comments from the reviewers:

Reviewer #1: Major Comments:

a) The MicroCOSM model is described in general terms in both the manuscript and supplementary materials, but key submodules (e.g., for education, income, sexual behaviour) lack sufficient detail for replication. I recommend including a summary table listing key inputs, outputs, and governing rules for each sub-model, possibly with illustrative pseudo-code or flow diagrams.

**RESPONSE**: We have followed the reviewer’s suggestion of adding further flow diagrams illustrating the model structure in Figure 1: panel b now shows the model of educational attainment, panel c shows the modelling of sources of household income, and panel a shows the model of sexual behaviour (see the amended Figure 1 below). Although we appreciate the reviewer’s request for more detail, all of the information is provided in the supplementary materials (see section 1.3 on modelling income and section 1.4 on modelling sexual behaviour) or in the previous MicroCOSM report [2] (section 3.5 describes the model of education and section 4 describes the model of sexual behaviour). We have added the following sentence in the Methods section: “*The modelling of educational attainment has been described previously [2], and is illustrated in Figure 1b.*”

Figure 1: Model structure

In panel (a), high-risk women can move in and out of sex work and high-risk men can engage in sex with sex workers at any time (not shown); only high-risk individuals can have more than one partner at a time. In panel (b), all individuals currently in school can leave school at any time (not shown), and return to school is permissible only in “School support” intervention scenarios. Panel (c) shows how eligibility to receive different types of income varies in relation to the age of each household member (“School support” and “Cash transfers” only apply in the relevant intervention scenarios). In panel (d), dashed lines represent ‘feedback effects’ of reproductive health outcomes on education and employment. LT = long-term (cohabiting/marital), ST = short-term, STI = sexually transmitted infection.

b) The model's assumptions regarding behavioural mechanisms (e.g., how socioeconomic status impacts condom use, sexual debut, concurrency) should be described with greater precision, including any evidence base supporting these pathways.

**RESPONSE**: These aspects are described in detail in the supplementary materials, and it is not clear if the reviewer is wanting us to move this justification into the main text, or if the reviewer is only looking for some mention of the relevant supplementary text in the main file. To summarize the relevant sections of the supplementary file:

- Section 1.4.1 explains the modelled effect of being in school on sexual debut, as well as the effect of household income on young females’ rates of sexual debut.
- Section 1.4.2 explains the modelled effect of household income on female entry into casual/ transactional sex, and the effect of employment on male entry into casual/transactional sex.
- Section 1.4.3 explains the effect of employment on male rates of contact with sex workers.
- Section 1.4.4 explains the effect of employment on male rates of short-term partner acquisition (and by extension, on male partner concurrency).
- Section 1.4.5 explains the effect of education on condom use.
- Section 1.4.6 explains the effect of education and being in school on rates of entry into marriage/cohabitation.

Our preference would be to not move the 24 pages that constitute section 1.4 of the supplementary materials into the main text, as this would add substantially to the length of the paper, and the detail would overwhelm most readers. We have instead added to the following sentence in the main text: “Table 1 summarizes the key parameters that relate socio-economic status to sexual risk behaviour and health seeking behaviour (*described more fully in sections 1.4 and 1.5 of the supplementary materials*).”

c) The combination of trial-derived and observational data for prior distribution specification is methodologically reasonable. However, several priors appear overly narrow or based on limited data, without adequate justification for the selected bounds. A clearer rationale for the shape and truncation of priors, supported by references, should be included in the supplementary materials.

**RESPONSE**: The supplementary materials provide detailed descriptions of the prior distributions and the evidence on which they are based (with a summary in Table S27), but we acknowledge that in some places the justification for the prior distributions was unclear, or the chosen parameters were arbitrary. We have revised the prior distributions, where appropriate, and have also provided more explanation in some cases. (Because of these changes to the prior distributions, the results in the paper have changed slightly.) To summarize the main points:

- The prior distribution for the increase in female sexual debut per log reduction in APCHI (adjusted per capita household income) is explained in section 1.4.1. We have added the following sentences: “*The odds ratios measured in the Zimbabwean and Cape Town studies do not provide an accurate measure of the K parameter, because the latter applies only at log income levels below the average, whereas the odds ratios are calculated across the full income/wealth range. If the parameter truly applies only below the average, but it is estimated in a regression model that includes the roughly half of individuals with log income values above the average, the regression coefficient will be diluted (shrunk towards zero by roughly a half).* Based on the available evidence, we represent the uncertainty in the *K* parameter using a gamma hurdle distribution with a weight of 0.1 given to the value of 0 (no effect) and a weight of 0.9 given to gamma distribution with mean *0.34 (twice the average of the Zimbabwean and Cape Town studies)* and standard deviation of *0.22 (twice the standard deviation of the same two studies)*.” Note that this is a small change from the values previously specified.
- The relative rate of female sexual debut if they are in school (compared to not in school) is explained in section 1.4.1. The mean of 0.4 for the non-hurdle component of the prior is the average estimate from a Malawian study [3] and two South African studies [4, 5]. The standard deviation of 0.23 is calculated from the same three studies, but we had neglected to mention this. We have modified the relevant sentence to read “…a beta distribution with a mean of 0.4 (the average of the values in the three previously cited studies) and standard deviation of 0.23 (*calculated from the same three studies*).”
- The relative rate of male sexual debut if they are in school (compared to not in school) is explained in section 1.4.1. The mean of 0.6 is based on two South African studies [4, 5], but we did not explain the standard deviation. We have modified the explanation: “…a beta distribution with a mean of 0.6 (the average of the values in the two previously cited studies) and standard deviation of 0.23 (*the same as for females due to the lack of male data*).”
- The prior distribution for the increase in female casual sex per log reduction in APCHI is explained in section 1.4.2. We showed that with the prior mean of 0.85, the ratio of casual sex at the 10^th^ percentile of household incomes to that at the 50^th^ percentile is 2.2, which is the average of the odds ratios in the South African studies [6-9]. Applying the same calculation to the individual studies, we estimate a standard deviation of 0.47. We have modified the text to explain the standard deviation: “Applying the same adjustment to the individual odds ratio estimates yields a standard deviation of 0.47, the same value as used for the prior distribution.” This is a small change from the standard deviation previously specified.
- The prior distribution for the increase in male casual sex if employed is explained in section 1.4.2. We have modified the relevant sentences: “…a gamma distribution with a mean of 0.63 and standard deviation of 0.42. The gamma mean corresponds to a relative risk of 1.63, *the average of* the estimates of 1.33 and 1.92 from the previously cited South African sources [9, 10], *and the standard deviation is calculated from the same two studies*.” This is a small change from the values previously specified.
- The prior distribution for the increase in male casual sex if employed is explained in section 1.4.3. We noted that the relative rate of 1.8 is the average of a South African study estimate [11] and a pooled analysis of African DHS data [12]. We have added the following sentence: “*In an analysis of 2016 South African DHS data (similar to that in the pooled analysis of African DHS data [12]), the effect of male employment on recent commercial sex activity was non-significant (aOR 1.16, 95% CI: 0.74-1.87), after controlling for age, marital status and urban location (author’s calculation).*” We have also modified the justification of the prior: “…a gamma distribution with a mean of 0.8 and standard deviation of 0.4. The mean of 0.8 corresponds to a relative risk of 1.8, which is consistent with both the South African study [11] and the analysis of African DHS data [12], *and the standard deviation was chosen such that the 2.5 percentile (0.2) would be consistent with the more conservative odds ratio of 1.2 from the South African DHS*.”
- The prior distribution for the effect of male employment on short-term partner acquisition is explained in section 1.4.4. We noted that the mean of 0.43 was the average from three data sources. We have modified the sentence to explain the standard deviation as well: “a gamma distribution with mean 0.43 (the average of the three studies that most directly measured the effect of male employment/income [9, 13, 14]) and standard deviation of 0.25 (*calculated from the same three studies*).”
- The prior distribution for the increase in consistent condom use per year of schooling is explained in section 1.4.5. We noted in this section the plausible range of 1-1.22 on the odds ratio parameter, based on comparing our model simulations of the relationship between condom use and education and those measured in the 1998, 2003 and 2016 Demographic and Health Surveys, and this was the basis for the prior distribution. (No change was made here.)
- The prior distribution for the relative rate of marriage if currently in school was explained in section 1.4.6. The prior distribution is based on the odds ratios estimated for males and females when fitting a logistic regression model to data from the 1993 October Household Survey. We have modified the prior description: “… a beta distribution with mean of 0.3 (*the average of the male and female odds ratios*) and standard deviation 0.2 (*calculated from the same two odds ratios*).”
- The prior distribution for the increase in medical male circumcision per log increase in APCHI is explained in section 1.5.2. The only data that could be used to estimate this parameter are from the Lesotho PHIA [15], but we argued that the estimate derived from these data (0.42) was an upper bound. We have modified the explanation of the prior: “To represent the uncertainty in the *C* parameter, we therefore assign a gamma prior distribution with a mean of 0.2 and a standard deviation of 0.1; *this was chosen such that the 97.5 percentile (0.44) was close to the upper bound estimated from Lesotho data, and the mean was roughly at the midpoint between 0 and the upper bound (i.e. allowing for a reasonably wide range of uncertainty around the parameter)*.”
- The prior distribution for the relative rate of school dropout if receiving school support (non-material support) is described in section 1.6. The mean of 0.68 is based on a systematic review of attendance monitoring interventions [16]. We did not previously provide much justification for the standard deviation, but we have provided more explanation in the updated description: “In a systematic review and meta-analysis of interventions to reduce school dropout , Wilson *et al* [16] estimated that attendance monitoring interventions significantly reduced the odds of school dropout (OR 0.68, 95% CI: 0.61-0.76); more significant effects were estimated for other types of dropout intervention, but these interventions were generally different from the school support interventions evaluated in African settings. A limitation of this review is that it reported almost exclusively on US data, *and the 95% confidence interval is therefore a poor reflection of the uncertainty around the outcome if the trial was conducted in a South African setting*. In addition, no estimate of the heterogeneity in attendance monitoring effects was provided. *However, the average OR, across all school dropout interventions, was 0.58 and the τ^2^ across all interventions (a measure of heterogeneity across individual trials, on a log scale) was 0.56. This τ^2^ statistic is equivalent to a standard deviation on the OR scale of approximately 0.39, but is likely to overstate the true standard deviation of random effects because it does not take into account that much of the variation is attributable to differences between intervention types. To represent the uncertainty in* *S*, we assign a beta prior with a mean of 0.68 and a standard deviation of *0.20. The mean is the same as the average effect of attendance monitoring interventions, while the standard deviation has been set to half of the estimated upper limit.* (This distribution has 2.5 and 97.5 percentiles at *0.24 and 0.97 respectively*.)” This is a small change from the standard deviation previously specified.
- The prior for the relative rate of school dropout per R800 increase in the ‘cash equivalent’ value of the school support is explained in section 1.6. The mean of 0.86 is based on the systematic review of Garcίa and Saavedra [17], but we did not previously explain the justification for the standard deviation. We have modified the explanation: “Garcίa and Saavedra [17] estimated that these interventions reduced the annual probability of secondary school dropout by an average of 2.9% (*with standard deviation of 2.1% across studies*). If the average annual probability of high school dropout is around 20% (as we have previously estimated in South Africa [2], and in line with settings in which high school dropout interventions are typically tested [16]), this 2.9% reduction is equivalent to a relative rate of dropout of 0.86 (with standard deviation of 0.10, or 0.021/0.2).”
- The prior for the effect of vocational training on unemployment is explained in section 1.6. The mean is based on the meta-analysis of Tripney *et al* [18], and the standard deviation was chosen such that the 97.5 percentile of the distribution would match the largest effect measured in any of the included studies. (No change was made here.)

d) The use of hurdle priors (Section 2.1 in supplementary file) to reflect uncertainty around null effects is innovative, but their functional form and probability mass allocation are not fully explained. A brief sensitivity analysis on the choice of hurdle probabilities would be valuable.

**RESPONSE**: The functional form and probability mass are explained in the first two equations in section 2.1 of the supplementary materials. We have expanded the example of the gamma hurdle distribution in section 2.1 of the supplementary materials. In this example, we consider the effect of household income (per log increase in household income) on the rate of medical male circumcision, noting that there was “some evidence from RCTs to suggest that an effect exists, but RCTs are not consistent or the evidence is considered weak” (category II evidence). When reviewing the observational data for this parameter, we estimated an upper limit of 0.42 (see section 1.5.2 of the supplementary material). For the purpose of illustration/sensitivity analysis, we consider four possible specifications of the prior:

1. The original gamma hurdle prior (using a weight of 0.25 for the hurdle)
2. An alternative gamma hurdle distribution, with a hurdle weight of 0.5 (category I evidence) and the same gamma component as the original
3. An alternative gamma hurdle distribution, with a hurdle weight of 0.1 (category III evidence)
4. A more conventional gamma prior with no hurdle, but with a somewhat lower mean (0.15) and higher standard deviation (0.11) to account for the uncertainty about whether there truly is an effect of household income on medical male circumcision (this has a 97.5 percentile of 0.43, similar to the upper limit noted previously).

These four prior distributions have similar means, and the same lower limit of zero, but with very different shapes; these are shown in Figure S13a. We further consider how the posterior distribution changes, assuming a single RCT data point in model calibration (the study of Cho *et al* [19] measured an effect of 0.507, with standard deviation of 0.248, as shown in Table S28). (This is a simplified example because the Cho study did not actually report by how much the intervention changed household incomes – we are implicitly assuming a unit change in the log of household incomes due to the intervention, which is probably unrealistic.) Figure S13b shows that the posterior distributions are similar at the upper end of their range, but quite different in the posterior weight given to a null association (as is appropriate given different levels of prior confidence in a true effect). Overall the posterior means are similar across the four prior specifications (0.192, 0.223, 0.236 and 0.210), despite the differences at the lower end of the posterior range.

Figure S13: Illustrative example of the use of different gamma hurdle distributions (on the proportionate increase in the rate of medical male circumcision, per log increase in household income)

The prior and posterior distributions are presented as the probability densities (y axis) over different ranges of the effect of household income on circumcision (x axis).

e) The Bayesian calibration approach is appropriate, but the likelihood construction is insufficiently described. Details should be added on how different trial outcomes were weighted in the composite likelihood, variance assumptions for the outcome measures, and any convergence diagnostics or posterior predictive checks performed.

**RESPONSE**: The likelihood construction is explained in section 2.2.4 of the supplementary materials. A likelihood value was calculated for each trial outcome separately; there was no ‘weighting’ applied in cases of multiple trial outcomes (effectively we are giving equal weight to different outcomes, and it is not clear that different weights would be necessary). We have added a sentence to clarify this point: “*Equal weights are given to all trial outcomes in the likelihood calculation.*” The variance assumptions are also explained in detail in section 2.2.4: we account for both measurement error (as reflected in the published 95% confidence intervals around the trial results), random effects (to account for variability in intervention impacts across different populations and intervention formulations) and stochastic error (to account for imprecision in the outputs of the agent-based model). As explained in section 2.3 of the supplementary materials, we use Sampling Importance Resampling (SIR) to generate the posterior distributions. This is a well-established Bayesian procedure [20], which does not require a convergence criterion, as it is not iterative/adaptive. However, there is the concern that the posterior sample might consist of only a few distinct parameter combinations (which can happen if only a very small region of the parameter space provides adequate fits to the data, and the prior distribution doesn’t efficiently sample from this region). To address this concern, we have added Table S29 in the supplementary materials showing the maximum posterior weight given to any single parameter combination and the number of distinct parameter combinations in the posterior sample. The table shows that no single parameter combination carries excessive weight in the posterior sample and we have an adequate mix of distinct parameter combinations. Although we have not performed posterior predictive checks, the simulated trial outcomes are compared to actual trial outcomes in Figures S14-S16 (the former generally have lower variance than the latter because the simulated outcomes don’t reflect the random effect terms assumed in the likelihood calculation). Even if we were to generate new simulated data sets and add random effect terms, it would be difficult to make statements about the validity of the posterior predictive distributions, given the sparseness of the actual RCT data for each individual outcome.

Table S29: Number of distinct parameter combinations and frequency of most frequently-sampled parameter combination in posterior sample of 100 parameter combinations

| Calibration data set | Distinct  parameter combinations | | Most frequently sampled  parameter combination | |
| --- | --- | --- | --- | --- |
|  | n | % | n | % |
| All RCT data | 82 | 82% | 7 | 7% |
| Vocational training RCTs | 99 | 99% | 2 | 2% |
| Cash transfer RCTs | 99 | 99% | 2 | 2% |
| School support RCTs | 90 | 90% | 6 | 6% |

f) Only 100 simulations were drawn from the posterior sample. This seems modest given the dimensionality of the model. The authors should comment on the adequacy of this sample size for uncertainty quantification and consider increasing it.

**RESPONSE**: We have doubled the posterior sample size, and the results have been updated to reflect this. However, we acknowledge that this only partially addresses the reviewer’s concern. We are constrained by the long time that it takes to run each simulation (and the need to run each simulation multiple times in order to reduce stochastic variation), which presents a formidable computing challenge. We believe that the mean estimates are relatively stable, and would not change much with a larger posterior sample, but the upper and lower limits on the 95% confidence intervals could change more materially with a larger posterior sample. We have added the following sentence when discussing the study limitations: “*Due to the substantial computing time required for each simulation, it was not feasible to draw a larger posterior sample, which would be important in obtaining more accurate 95% confidence intervals. The 95% confidence intervals should therefore be interpreted with caution.*”

g) The assumptions underlying the implementation of interventions (e.g., universal access, full adherence, constant effect sizes) may not reflect real-world feasibility. I recommend including more realistic scale-up scenarios or at least discussing this as a limitation.

**RESPONSE**: We assume that the reviewer is referring to the second part of the results (where we consider the potential future effect of introducing economic strengthening interventions nationally), not the initial results from the model calibration (for the purpose of simulating randomized controlled trials, we believe it would be reasonable to assume a high level of fidelity to the intervention). We agree that some of the assumptions made when simulating the future rollout of economic strengthening are overly optimistic, but this was intentional: we aimed to assess what might be achieved in a ‘best case scenario’. The results are non-significant despite the optimistic assumptions, and this reinforces the point that the interventions are unlikely to have much impact in a ‘real world’ setting. We have added a sentence to the Discussion stressing this point: “*When modelling the future rollout of economic strengthening interventions, we have optimistically assumed that all eligible individuals would receive the intervention and achieve levels of exposure similar to those in RCTs. This assumption is unrealistic, but the non-significance of the simulated intervention impacts, despite the optimistic assumptions, reinforces the point that they are unlikely to have substantial impacts in the South African setting.*”

h) The counterfactual scenario—where all individuals attain tertiary education, secure employment, and earn above the national income average—appears implausibly optimistic. This may bias the estimated population attributable fractions (PAFs) upward. A more conservative or gradient-based counterfactual would improve credibility.

**RESPONSE**: We agree that the counterfactual scenario is unrealistic. However, counterfactual scenarios are not generally supposed to be realistic, and in an epidemiological context, when calculating a PAF, it is common for the counterfactual to be highly unrealistic. For example, in assessing the PAF for the burden of disease due to physical inactivity, it is commonly assumed in the counterfactual scenario that everyone in the population (including the elderly and disabled) has >1000 minutes of vigorous physical activity per week [21]. And in assessing the PAFs for sugar sweetened beverages and processed meats it is assumed that consumption of both would reduce to zero in the counterfactual, even though such a change would be virtually impossible to achieve in reality, at a population level [22].

i) The model treats changes in socioeconomic status.as exogenous to behavioural risk, but real-world interactions (e.g., pregnancy affecting education, HIV status affecting employment) suggest bidirectional causality. These feedbacks are not captured in the current model and should be acknowledged explicitly.

**RESPONSE**: Our model does incorporate the feedback effects to which the reviewer refers, and both feedback effects are represented in Figure 1d (by the arrow from the HIV incidence outcome to the unemployment outcome, and by the arrow from the teenage pregnancy outcome to the incomplete schooling outcome). We have added a legend to Figure 1 explaining that these arrows represent feedback effects. The effect of HIV on employment is described in more detail in section 1.1.2 of the supplementary materials (and is also briefly explained in the third paragraph of the Methods section of the main text). The effect of teenage pregnancy on education was described in more detail in an earlier report on the model [2], but we have added the following text in the footnote to Figure 1: “*As described previously [2], girls who fall pregnant while in school are assumed to either drop out of school permanently or to repeat the grade they were in at the time of the pregnancy.*”

j) Trial-derived effect estimates are applied nationally, but trials such as HPTN 068 and Swa Koteka may not reflect national demographic or geographic variation. The authors should address how they accounted for the limited generalisability of trial populations in model calibration.

**RESPONSE**: The focus here is on the heterogeneity in effects across settings. We agree that this is a concern, and we addressed this by including a random effect term in the likelihood function (as explained in response to comment e). It is also worth repeating that the interventions are assumed to be limited to people whose average per capita household incomes are below the national average, which partially addresses the concern that the trials are usually conducted in very impoverished populations.

k) While the model is validated against national survey data, reliance on cross-sectional associations is limiting. Additional external validation (e.g., longitudinal data, cohorts not used in calibration) would improve credibility.

**RESPONSE**: We have added the following sentence in the discussion of limitations: “*Our analysis relies only on publicly available data, but additional longitudinal individual-level data, such as collected in Health and Demographic Surveillance Systems (HDSSs) [23], may be valuable in validating the model assumptions about effects of socio-economic status on sexual behaviour, fertility and HIV.*” Although we agree with the reviewer, such an analysis would require substantial additional work, as data from the HDSSs are typically not publicly available, and need to be requested, together with appropriate ethical approval. Even if the data are easily accessible, there is the concern that these HDSSs are typically not nationally or regionally representative (for example, most of the HDSSs are located in rural settings), which limits generalizability, and it would probably be inadvisable to rely on data from a single HDSS. Given the time frame allowed for resubmission, a more extensive validation of the model against longitudinal data is not feasible.

l) The authors may also wish to discuss whether their findings are transferable to other sub-Saharan African contexts, and under what conditions such generalisation might be appropriate.

**RESPONSE**: We have acknowledged that South Africa differs from other African settings: “Our results might not be generalizable to other African settings, where there is typically less income inequality but more absolute poverty.” Countries like Namibia and Botswana are more similar to South Africa in socioeconomic terms (high income inequality but low average poverty levels), whereas countries like Mozambique and Malawi are very different from South Africa. We have also added the following sentence in the same paragraph: “*In a recent analysis of national household survey data, HIV prevalence was inversely associated with education in southern and East Africa, but not in West and Central Africa [24]. Unlike in southern Africa, ART coverage and socioeconomic status were positively associated in other African regions [24].*”

m) The model code is stated to be available upon acceptance. In line with open science standards, I strongly recommend that the code, calibration data, and simulation instructions be made available at the time of peer review to enable full reproducibility.

**RESPONSE**: We have made the code available on Github at <https://github.com/leighjohnson/MicroCOSM>. This includes the calibration data (in the CashTransferRCTs, SchoolSupportRCTs.txt and VocationalTrainingRCTs files, which contain the same data as in Table S28 in the supplementary materials) and a Readme file that explains how to run the model. We have added the following sentence in the main text: “*All model code and data inputs are available from* [*https://github.com/leighjohnson/MicroCOSM*](https://github.com/leighjohnson/MicroCOSM)*.*”

Minor Comments:

n) The adjusted per capita household income (APCHI) scale is used without discussion of alternatives (e.g., OECD-modified scale).

**RESPONSE**: There are several equivalization methods, such as the original OECD scale and the OECD-modified scale to which the reviewer refers. We have added the following comment at the end of the paragraph in which the APCHI is defined: “*Other equivalence scales are reviewed elsewhere [25].*” We have also added a mention of alternative equivalence scales in the first paragraph of section 1.3 of the supplementary materials.

o) Income is modelled as relatively stable, which may understate income volatility and its effects on behaviour. If dynamic income variation is not modelled, this should be acknowledged.

**RESPONSE**: Income is modelled dynamically: it changes as individuals move in and out of employment, and as they qualify for social grants (or lose eligibility). At the household level income can also change due to changes in household composition (e.g. loss of a breadwinner, or addition of a child that qualifies the caregiver to receive a Child Support Grant). This dynamic modelling of income sources is described in section 1.3 of the supplementary materials. We have added the following sentence at the start of section 1.3 (in the third paragraph) to clarify this point: “*Household income is re-calculated at annual time steps, taking into account changes in household composition, employment and social grant eligibility.*”

p) It is unclear how the model handles uncertainty in parameters derived from heterogeneous or sparse data sources. A table mapping source type to uncertainty specification would improve transparency.

**RESPONSE**: Table 1 lists the most important parameters and the data sources on which they are based; where a standard deviation is specified, the parameter is varied in the model calibration/Bayesian analysis. For all other parameters, the value is fixed. To make this clearer, we have added a footnote to Table 1 explaining this point: “*Standard deviations are specified for the parameters that are varied in the model calibration process (sections 1.4-1.6 of the supplementary materials describe the prior distributions in more detail); for all other parameters, the value is fixed.*” There are hundreds of parameters in this model, most of which are not central to our research question, and we only assess the uncertainty in the parameters that are most relevant to the current research question. To investigate/explore the uncertainty in all parameters would be infeasible.

q) Behavioural outcomes (e.g., concurrency, condom use) may be sensitive to social network structures and local norms, which are not accounted for in the current individual-level modelling. This could be discussed as a potential limitation.

**RESPONSE**: The behavioural variables referred to are modelled as being determined by gender norms, which vary in relation to urbanization, age and race – so there is an implicit modelling of variation of local norms and their influence on behaviour. This is described in more detail in our previous publication on the modelling of inequitable gender norms and their effects on sexual behaviour [1]. However, the reviewer is correct in pointing out that the model doesn’t explicitly represent the effect of social networks on behaviour, which is likely to be significant [26]. We have added the following sentence in the third-last paragraph to acknowledge this: “*The model also does not represent the effect of social networks on sexual behaviour [26], and the formation of these networks is likely to be heavily influenced by education and employment.*”

r) Time horizon of 15 years may be insufficient to capture the full long-term or intergenerational benefits of economic interventions, especially those affecting educational attainment. This limitation should be acknowledged.

**RESPONSE**: We agree, and have added the following sentence in the Discussion: “*The model does not represent intergenerational transfers in education [27], and a 15-year projection term is likely to be insufficient for representing the longer-term benefits of investment in education.*” However, it is worth noting that we did not find evidence of impacts increasing or decreasing over the 15-year projection term, and we have added section 3.4 in the supplementary materials to demonstrate this more clearly.

Reviewer #2: This manuscript presents a valuable effort to model the complex relationship between economic strengthening interventions and HIV, STIs, and teenage pregnancy in South Africa. The use of an agent-based model (MicroCOSM) is commendable, and the study attempts to quantify the potential impact of various interventions. However, several aspects require further clarification and justification. The limited impact of the modeled interventions is a significant finding, but the reasons behind this need to be explored more thoroughly.

1. "Over 2025-2040, none of the interventions are estimated to have impacts significantly different from zero, due to limited impact on secondary economic outcomes." This is not clear and misleading. What are the interventions? The methods part should highlight this.

**RESPONSE**: The three interventions were defined in the paragraph before Table 1 (in the Methods section). To reinforce the point, we added the following sentence in the final paragraph of the Methods section: “*Three future intervention scenarios were modelled: school support, vocational training and cash transfers.*”

2. The wide confidence intervals for the PAF estimates should be highlighted as a key limitation.

**RESPONSE**: This limitation is acknowledged in the first sentence of the Discussion: “Although our model suggests a potentially significant contribution of low socio-economic status to the incidence of HIV, STIs and teenage pregnancy in South Africa, there is substantial uncertainty around the magnitude of this contribution.”

3. The rationale for using a modeling approach should be clearly stated. What specific questions can the model address that cannot be answered through empirical studies alone?

**RESPONSE**: We have added a few sentences in the Introduction to make the rationale clearer. “*They* [mathematical models] *also play an important role in translating evidence from randomized trials, which produce short-term estimates of impact at an individual or community level, into estimates of likely longer-term impact at a population level…. Agent-based models, which represent individual-level variation in health risk exposure and outcomes, are well suited to representing complex causal pathways and diverse outcomes, and are increasingly utilized in assessing socio-economic disparities in health [28].*”

4. The introduction should also emphasize what gaps this modeling study aims to fill and how it builds upon existing literature.

**RESPONSE**: We previously noted in the Background that “there is a notable lack of mathematical modelling of economic strengthening interventions [29]. This is partly a reflection of the challenges associated with representing the complex causal pathways described previously, a challenge common to the modelling of structural interventions generally [30].” Although there are few relevant modelling studies in the context of reproductive health specifically, we have added to the Background by citing a review of relevant models of other health conditions: “*Building upon recent advances in the use of agent-based models to represent socio-economic differences in health outcomes [28], we aim to assess which economic strengthening interventions are likely to be most impactful*.” In the third paragraph of the Background, we also previously noted the lack of consensus on which economic strengthening interventions are most effective. We have added a sentence in this paragraph: “*Data from randomized controlled trials (RCTs) have not been systematically synthesized using traditional meta-analytic techniques, a reflection of the heterogeneity in interventions and trial outcomes.*”

5. The complexity of education and social economics on HIV and STIs may be relevant to different factors, i.e., rural or urban, and gender. The modeling should also take these into account, especially given no clear conclusions between those with HIV and STIs.

**RESPONSE**: We have added the following sentence in the Methods section: “*The modelling of educational attainment has been described previously [2], and is illustrated in Figure 1b.*” The model does allow for relationships between socio-economic status and urban-rural location/migration. As described in the cited report [2], people in rural areas are more likely to migrate to urban areas if they have higher educational attainment. In the cited report, we also validated the model assumptions about urban-rural differences in sexual behaviour by comparing the modelled estimates of urban-rural differences in HIV prevalence against published data [2]. We assume that people in urban areas are more likely to get employed (see section 1.1.1 in the supplementary materials of our submission). Similarly, the model allows for gender differences, with females having higher rates of grade advancement [2] but lower employment probabilities (and lower salaries) than their male counterparts, as described in sections 1.1.1 and 1.3.1 of the supplementary materials. We have also emphasized, throughout the paper and the supplementary materials, that the effects of socio-economic status on sexual behaviour are not the same in men and women.

6. What are the key assumptions of the model? How were these assumptions validated? The results section should report the validation results.

**RESPONSE**: The key assumptions (relevant to the current paper) are summarized in Table 1, and the more detailed assumptions are described in section 1 of the supplementary materials. The validation is described in detail in section 3.1 of the supplementary materials, and the results of these validations are briefly reported at the end of the second paragraph of the Results section. Some of the plots in Figure 2 can also be considered validations; for example, panels g and h show that the model is in good agreement with median income levels and Gini coefficient estimates (even though these were not used in model calibration). We have modified the sentence in the main text describing these results to make it clear that this is a validation: “The model also estimated rising income together with declining income inequality, the result of increasing levels of social welfare expenditure, *and both trends were validated by estimates from Statistics South Africa* (Figures 2g-h).” Panel b of Figure 2 shows that the model estimates of HIV incidence were also consistent with survey-based estimates of incidence (again, not used in calibration). Although it would be possible to move the validation plots from section 3.1 of the supplementary materials into the main text, our preference would be to not make this change, as the Results section is already quite long. We are willing to reconsider if the editor feels the rearrangement would be of value.

7. A flow diagram illustrating the key components of the model and their interactions would be helpful.

**RESPONSE**: We have expanded Figure 1 to provide more detail on the model structure. In addition to panel d (the previous Figure 1, which summarizes the causal pathways), we have added panel a to illustrate the modelling of sexual behaviour, panel b to illustrate the modelling of educational attainment, and panel c to illustrate how sources of household income vary in relation to household members’ ages. The updated Figure is shown in the response to the first comment from Reviewer 1.

8. The reliance on publicly available data is understandable, but the limitations of this data should be acknowledged.

**RESPONSE**: We have added the following sentence in the discussion of limitations: “*Our analysis relies only on publicly available data, but longitudinal individual-level data, such as collected in Health and Demographic Surveillance Systems (HDSSs) [23], may be particularly valuable in validating the model assumptions about effects of socio-economic status on sexual behaviour, fertility and HIV.*”

9. The process of calibrating the model to RCT data needs to be described in more detail. How were the various RCTs selected? How were the intervention effects translated into model parameters? What measures were taken to address heterogeneity across the RCTs?

**RESPONSE**: The calibration process is described in detail in section 2.2 of the supplementary materials. Section 2.2.1 explains how RCTs were identified from recent reviews. Section 2.2.4 describes in statistical terms how the likelihood was defined, including the random effects to represent heterogeneity across RCTs. The intervention effects in the RCTs are not “translated into model parameters” but rather compared against the model simulation of the trial outcome that might be expected, given a particular set of model input parameters, and the likelihood is calculated as a measure of the agreement between simulated and observed trial outcomes. This is described, together with an example, in the first two paragraphs of section 2.2.4 of the supplementary materials.

10. The rationale for using hurdle distributions for the socio-economic status parameters should be explained more fully.

**RESPONSE**: We have added the following in the main text: “*this* [hurdle] *was included to avoid excessive prior confidence in the purported causal relationship*”. The rationale for using hurdle distributions is further explained at the start of section 2.1 of the supplementary materials. But to make the rationale clearer, we have added the following sentence after the introduction of the gamma prior: “*If we were not to include a hurdle (i.e. set θ = 0, to give a more conventional gamma prior), we would be excluding the possibility of a null association, which would be overly optimistic.*” We have also added an example of possible alternative prior specifications, and demonstrated how the selection of prior (and hurdle) can influence the posterior estimates (see response to point d by the first reviewer).

11. The choice of the three intervention types (school support, vocational training, and unconditional cash transfers) needs to be justified. Are these the most promising or widely implemented economic strengthening interventions in South Africa?

**RESPONSE**: We have added the following sentence in the second paragraph of the ‘Model calibration’ section of the Methods: “*Less frequently studied interventions (e.g. savings programmes, food assistance, financial education) were excluded due to limited RCT data available on their effects [31].*” This question was also partially answered in the second paragraph of section 2.2.1 of the supplementary materials: “We also excluded studies of savings programmes, if these did not include elements of vocational training [32], and lottery-based interventions [33], in both cases because the hypothesized effect was on future wealth rather than short-term socio-economic improvement.”

12. The assumptions about how the interventions affect model parameters should be clearly stated.

**RESPONSE**: We have added a couple of sentences and modified the sentence describing the modelling of school support, in the paragraph before Table 1: “The school support intervention is assumed to include both material support (e.g. school uniforms, cost of school transport) and non-material support (e.g. attendance monitoring and counselling), *both of which reduce the probability of school dropout*…. *Vocational training is assumed to increase the odds of finding employment in currently unemployed individuals.* *Cash transfers are assumed to be added to total household income (another potential source of income).*” The relevant parameters are also shown in Table 1.

13. The methods for calculating PAFs are described.

**RESPONSE**: It is not clear if this was a typo (and the reviewer meant ‘not described’), or if the reviewer was merely noting this for completeness. The methods for describing the PAF calculation are described in the second-last paragraph of the Methods section. To make things clearer, we have changed the previous ‘Model scenarios’ heading to ‘Population attributable fractions’ and added a heading before the last paragraph: ‘Future intervention scenarios’.

14. The finding that none of the interventions have a significant impact over 2025-2040 is surprising. The reasons for this lack of impact should be explored in more detail. Is it due to the limited reach of the interventions, the complexity of the causal pathways, or limitations in the model? In the methods section, the author should mention the impact of the selected interventions from previous results/models.

**RESPONSE**: The lack of impact is likely to be due to all three of the factors that the reviewer mentions (or at least the first two – it is difficult to pinpoint specific issues in the model structure that would cause a lack of input). We showed in Figure 3 that the interventions have only a modest impact on socioeconomic indicators, and we have added further detail to Table 4 showing that that the interventions have almost zero impact on intermediate behavioural outcomes (see the response to the editor’s first comment). When one is dealing with long, complex causal pathways, the intervention impact at the first step in the causal pathway gets diluted at each subsequent step in the pathway. We have added this sentence in the Discussion: “*These modest effects on socioeconomic indicators are further diluted at each subsequent step in the causal pathway, so that the final effects on reproductive health outcomes are negligible.*” As we noted in the Introduction, there have been no detailed previous models of the impact of economic strengthening interventions on HIV, so it is difficult to incorporate these in the Methods, as the reviewer suggests. However, we have mentioned the results of a relatively simple Brazilian modelling study [34, 35] in the Discussion, and the last column in Table 1 references the results of previous studies that inform our choice of model assumptions about the intervention effects.

15. The discussion should also consider the policy implications of the findings. If economic strengthening interventions are unlikely to have a significant impact on HIV, STIs, and teenage pregnancy, what other strategies should be pursued?

**RESPONSE**: It is difficult to identify clear policy implications. We would argue that in the current context, where HIV funding is still ‘ringfenced’ but increasingly constrained, it makes sense to focus HIV spending on interventions that have been shown to be highly cost-effective, e.g. condom distribution, ART and PrEP for key populations [36]. However, in the longer term it makes sense to move away from this ‘siloed’ way of thinking about budgets and programmes, and evaluate the value of economic strengthening more holistically, considering the gains across diverse domains, similar to work pioneered by Remme *et al* [37] on using cross-sectoral cost-benefit analysis to guide funding allocation across health and other sectors for structural interventions. We see this as a long-term goal both because we currently lack the appropriate analytic tools and because current funding streams are still very disease-specific. We had hinted at this previously in the final paragraph, but have amended this to make it clearer. The final paragraph now starts: “*In the short term, and in the context of a dedicated but declining budget for HIV programmes, it would make sense to focus HIV spending on interventions that have been shown to be highly cost-effective in reaching HIV endpoints (e.g. condom distribution, ART and pre-exposure prophylaxis for key populations [36]), rather than economic strengthening. However,* economic interventions can have important positive impacts beyond sexual and reproductive health, including on mental health [19, 38-40], tuberculosis [41], child health [42] and social well-being [43]. Ideally, all of these outcomes should be considered when evaluating the impact or cost-effectiveness of economic interventions, and we do not suggest that policy decisions should be based on sexual and reproductive health outcomes alone.”

16. The authors need to acknowledge that social and environmental factors will also contribute to social wellbeing.

**RESPONSE**: The comment is not completely clear, but we assume the reviewer is referring to social well-being as distinct from health, and the positive relationship between education and social well-being [43]. We have modified the sentence in the final paragraph of the Discussion: “Economic interventions can have important positive impacts beyond sexual and reproductive health, including on mental health [19, 38-40], tuberculosis [41], child health [42] *and social well-being* [43].”

Reviewer #3: Leigh Johnson and colleagues used a previously developed agent based model to investigate the role of socioeconomic factors that drive South Africa's high incidence rates of HIV, STI and teenage pregnancies. Examining these socioeconomic factors within a model is important due to the complex relationships between these socioeconomic factors. The agent-based model that was used included the effects of education, employment and per-capita household income on sexual behaviours. Using the model the authors found that that low socioeconomic status accounted for 14% of new HIV infections, 10% of STIs, and 46% of teenage births from 2000 to 2020. However, because of uncertainties regarding effect sizes, confidence intervals around these PAFs are wide. Over 2025-2040, none of the interventions are estimated to have impacts significantly different from zero, due to limited impact on secondary economic outcomes. The authors conclude that although povery is a significant driver of HIV, STIs and teenage pregnancy, precise quantification is challenging. In addition, extrapolating economic strengthening interventions to daily practice is expected to have insufficient impact on socioeconomic status to reduce HIV and STIs significantly at a population level.

The paper addresses a very important topic. I congratulate the authors on a well-designed and performed study. The article is well written. I have one minor comment: please add the national per-capita income in the asbtract as this is an important factor in the analysis.

**RESPONSE**: It is difficult to integrate this detail in the abstract. However, we have modified the following sentence in the Background to include the per-capita GDP: “*Although South Africa has a high gross domestic product per capita, by African standards ($6253 in 2024 [44]), it also has* the highest level of income inequality globally [45], *and is therefore an important setting in which to assess the relationship between socioeconomic status and reproductive health.*”

**Responses to general technical guidance**

* We ask every co-author listed on the manuscript to fill in a contributing author statement, making sure to declare all competing interests. If any of the co-authors have not filled in the statement, we will remind them to do so when the paper is revised. If all statements are not completed in a timely fashion this could hold up the re-review process. If new competing interests are declared later in the revision process, this may also hold up the submission. Should there be a problem getting one of your co-authors to fill in a statement we will be in contact. Please do not add or remove authors without first discussing this with the handling editor. You can see our competing interests policy here: http://journals.plos.org/plosmedicine/s/competing-interests.

**RESPONSE**: All contributing author statements have been submitted.

* Please upload any figures associated with your paper as individual TIF or EPS files with 300dpi resolution at resubmission; please read our figure guidelines for more information on our requirements: http://journals.plos.org/plosmedicine/s/figures. While revising your submission, please upload your figure files to the PACE digital diagnostic tool, https://pacev2.apexcovantage.com/. PACE helps ensure that figures meet PLOS requirements. To use PACE, you must first register as a user. Then, login and navigate to the UPLOAD tab, where you will find detailed instructions on how to use the tool. If you encounter any issues or have any questions when using PACE, please email us at PLOSMedicine@plos.org.

**RESPONSE**: All figures have been submitted as separate TIF files.

* Please ensure that the paper adheres to the PLOS Data Availability Policy (see http://journals.plos.org/plosmedicine/s/data-availability), which requires that all data underlying the study's findings be provided in a repository or as Supporting Information. For data residing with a third party, authors are required to provide instructions with contact information (web or email address) for obtaining the data. Please note that a study author cannot be the contact person for the data. PLOS journals do not allow statements supported by "data not shown" or "unpublished results." For such statements, authors must provide supporting data or cite public sources that include it.

**RESPONSE**: The data on which this study are based are publicly available, and have been referenced appropriately. We have also provided the data from randomized trials, which were used in calibration, in Table S28 of the supplementary materials. We have replaced the previous reference to “results not shown” with a reference to additional results in the supplementary materials. The revised sentence in the main text reads “Proportionate reductions in HIV, STIs and teenage pregnancies are also similar over the 2025-2040 period, i.e. there is no suggestion of the intervention impact waning or improving at longer durations (*Table S32*).” We have also added section 3.4 in the supplementary materials, which includes Table S32 and provides more detail on the tests of trend.

* We expect all researchers with submissions to PLOS in which author-generated code underpins the findings in the manuscript to make all author-generated code available without restrictions upon publication of the work. In cases where code is central to the manuscript, we may require the code to be made available as a condition of publication. Authors are responsible for ensuring that the code is reusable and well documented. Please make any custom code available, either as part of your data deposition or as a supplementary file. Please add a sentence to your data availability statement regarding any code used in the study, e.g. "The code used in the analysis is available from Github [URL] and archived in Zenodo [DOI link]" Please review our guidelines at https://journals.plos.org/plosmedicine/s/materials-software-and-code-sharing and ensure that your code is shared in a way that follows best practice and facilitates reproducibility and reuse. Because Github depositions can

be readily changed or deleted, we encourage you to make a permanent DOI'd copy (e.g. in Zenodo) and provide the URL.

**RESPONSE**: We have made the code available on Github at <https://github.com/leighjohnson/MicroCOSM>. This includes the calibration data (in the CashTransferRCTs, SchoolSupportRCTs.txt and VocationalTrainingRCTs files, which contain the same data as in Table S28 in the supplementary materials) and a Readme file that explains how to run the model. We have added the following sentence in the main text: “*All model code and data inputs are available from* [*https://github.com/leighjohnson/MicroCOSM*](https://github.com/leighjohnson/MicroCOSM)*.*”

* Please ensure that the study is reported according to the appropriate guideline and include the completed checklist as Supporting Information. When completing the checklist, please use section and paragraph numbers, rather than page numbers. Please add the following statement, or similar, to the Methods: "This study is reported as per [XXXX] guideline (S1 Checklist)."

**RESPONSE**: We note that there are no specific reporting guidelines for modelling studies, although the editors make some recommendations for modelling studies. We have responded to these points below, but have not submitted a separate ‘checklist’.

FORMATTING - GENERAL

* Abstract: Please structure your abstract using the PLOS Medicine headings (Background, Methods and Findings, Conclusions). Please combine the Methods and Findings sections into one section.

**RESPONSE**: The abstract is correctly structured.

* At this stage, we ask that you include a short, non-technical Author Summary of your research to make findings accessible to a wide audience that includes both scientists and non-scientists. The Author Summary should immediately follow the Abstract in your revised manuscript. This text is subject to editorial change and should be distinct from the scientific abstract. Ideally each sub-heading should contain 2-3 single sentence, concise bullet points containing the most salient points from your study. In the final bullet point of 'What Do These Findings Mean?', please include the main limitations of the study in non-technical language. Please see our author guidelines for more information: https://journals.plos.org/plosmedicine/s/revising-your-manuscript#loc-author-summary.

**RESPONSE**: We have added an Author Summary.

* Please express the main results with 95% CIs as well as p values. When reporting p values please report as p<0.001 and where higher as the exact p value p=0.002, for example. Throughout, suggest reporting statistical information as follows to improve clarity for the reader "22% (95% CI [13%,28%]; p</=)". Please be sure to define all numerical values at first use.

**RESPONSE**: We have only reported 95% CIs. This is a modelling study, and we are not attempting to test specific hypotheses, but rather to quantify the potential impact of different interventions, and the contribution of different risk factors. Given the objectives and study design, p values are in most cases unnecessary.

* Please include page numbers and line numbers in the manuscript file. Use continuous line numbers (do not restart the numbering on each page).

**RESPONSE**: We have done this.

* Please cite the reference numbers in square brackets. Citations should precede punctuation.

**RESPONSE**: We have done this.

FIGURES AND TABLES

* Please provide titles and legends for all figures and tables (including those in Supporting Information files).

**RESPONSE**: We have done this. However, we are unsure about the preferred approach for the supplementary materials. Currently there is one supplementary file, which includes 20 figures and 32 tables. We assume that the editor would prefer that we just list the one supplementary file at the end of the manuscript, rather than list all 52 figures and tables in the supplementary materials. This can be modified if the editor would prefer we include the more complete list.

* Please define all abbreviations used in each figure/table (including those in Supporting Information files).

**RESPONSE**: We have done this.

* Please consider avoiding the use of red and green in order to make your figure more accessible to those with color blindness.

**RESPONSE**: None of the figures include red-green combinations.

SUPPLEMENTARY MATERIAL

* Please note that supplementary material will be posted as supplied by the authors. Therefore, please amend it according to the relevant comments outlined here.

* Please cite your Supporting Information as outlined here: https://journals.plos.org/plosmedicine/s/supporting-information

**RESPONSE**: We have cited the supplementary material correctly. All references to the supplementary materials now include ‘S1 Text’ (the file in which all supplementary materials are included).

REFERENCES

* PLOS uses the numbered citation (citation-sequence) method and first six authors, et al.

**RESPONSE**: We have followed the required format.

* Please ensure that journal name abbreviations match those found in the National Center for Biotechnology Information (NCBI) databases (http://www.ncbi.nlm.nih.gov/nlmcatalog/journals), and are appropriately formatted and capitalised.

**RESPONSE**: We have used the correct abbreviations for journal titles in the reference list.

* Where website addresses are cited, please include the complete URL and specify the date of access (e.g. [accessed: 12/06/2023]).

**RESPONSE**: We have added the URLs and dates accessed, where relevant.

* Please also see https://journals.plos.org/plosmedicine/s/submission-guidelines#loc-references for further details on reference formatting.

**RESPONSE**: We have formatted all references correctly. We have included PMID numbers where relevant, although it was not clear from the instructions if these were necessary.

MODELLING STUDIES

The following list is derived from Geoffrey P Garnett, Simon Cousens, Timothy B Hallett, Richard Steketee, Neff Walker. Mathematical models in the evaluation of health programmes. (2011) Lancet DOI:10.1016/S0140-6736(10)61505-X:

* If pertinent, please provide a diagram that shows the model structure, including how the natural history of the disease is represented, the process and determinants of disease acquisition, and how the putative intervention could affect the system.

**RESPONSE**: This is included in Figure 1.

* Please provide a complete list of model parameters, including clear and precise descriptions of the meaning of each parameter, together with the values or ranges for each, with justification or the primary source cited and important caveats about the use of these values noted.

**RESPONSE**: We have done this in Table 1. It is perhaps worth noting that there are many hundreds of parameters in the model, and it is not feasible to detail all of them, but Figure 1 focuses on the parameters most relevant to the current study. Other parameters are explained in the supplementary materials or in previous published description of the model, which we have cited throughout our submission [2, 46, 47].

* Please provide a clear statement about how the model was fitted to the data, including goodness-of-fit measure, the numerical algorithm used, which parameter varied, constraints imposed on parameter values, and starting conditions.

**RESPONSE**: A brief summary of these aspects is provided in the main text: the ‘Model calibration’ section in the Methods summarizes the Bayesian calibration approach and the likelihood calculation (based on the log difference between the modelled and observed trial outcomes), and Table 1 shows the parameters that were varied in the calibration process. In the supplementary materials we provide more detail: section 2.2 describes the likelihood calculation, section 2.3 describes the numerical algorithm (Sampling Importance Resampling) and the constraints imposed on parameters are shown in section 2.1 (in the case of gamma priors, any positive value is permissible, and in the case of beta priors, any value between 0 and 1 is permissible). The starting conditions for the socio-economic parameters are described in sections 1.1.3 and 1.2.2 of the supplementary materials.

* For uncertainty analyses, please state the sources of uncertainties quantified and not quantified [can include parameter, data, and model structure].

**RESPONSE**: We have evaluated parameter uncertainty, and the specific parameters for which we consider uncertainty are shown in Table 1 (as indicated in the footnote, wherever a standard deviation (SD) is specified, this represents a parameter that is varied in the model calibration and uncertainty analysis). We do not consider uncertainty regarding the model structure; structural uncertainty would normally be represented by the comparison of different models, but given that this is a novel area, there are no other models that we can compare against. Uncertainty in the data used in calibration (the randomized controlled trial data) is not evaluated explicitly, but by using a random effect model in the specification of the likelihood, we are implicitly allowing for uncertainty regarding the applicability of the data in the South African setting.

* Please provide sensitivity analyses to identify which parameter values are most important in the model. Uncertainty estimates seek to derive a range of credible results on the basis of an exploration of the range of reasonable parameter values. The choice of method should be presented and justified.

**RESPONSE**: In Table 3 we use partial rank correlation coefficients (PRCCs) to show which parameters are most critical to the variation in population attributable fractions, and in the last paragraph of the Results we present similar PRCCs for the intervention impacts. We have also used Bayesian calibration techniques, which are appropriate to determining uncertainty ranges around all model outputs. Both aspects are described in the Methods.

* Please discuss the scientific rationale for the choice of model structure and identify points where this choice could influence conclusions drawn. Please also describe the strength of the scientific basis underlying the key model assumptions.

**RESPONSE**: We have amended the Background section to clarify the rationale for using a modelling approach: “*They* [models] *also play a role in translating evidence from RCTs, which produce short-term estimates of impact at an individual or community level, into estimates of likely longer-term impact at a population level.*” We also added a sentence to explain the rationale for an agent-based model specifically: “*Agent-based models, which represent individual-level variation in health risk exposure and outcomes, are well suited to representing complex causal pathways and diverse outcomes, and are increasingly utilized in assessing socio-economic disparities in health [28].*” In the Discussion, we have noted the limitation that we have not modelled the influence of income inequality at a community level, and this may have led to us understating the impacts of economic strengthening. We have been rigorous in setting the prior distributions in such a way that they reflect the uncertainty around how much of the observed associations between socioeconomic status and sexual behaviour are truly causal relationships – this is described in section 2.1 of the supplementary materials. In the Discussion, we have also stressed the uncertainty regarding the socio-economic effects on behavioural parameters, and the resulting wide confidence intervals around the model estimates.

* For studies that develop a prediction model or evaluate its performance, please ensure that the study is reported according to the TRIPOD statement (https://www.equator-network.org/reporting-guidelines/tripod-statement) and include the completed checklist as Supporting Information. Please add the following statement, or similar, to the Methods: "This study is reported as per the Transparent Reporting of a Multivariable Prediction Model for Individual Prognosis Or Diagnosis (TRIPOD) statement (S1 Checklist)." For studies using machine learning, please use the TRIPOD-AI checklist. When completing the checklist, please use section and paragraph numbers, rather than page numbers.

**RESPONSE**: This is not applicable to our submission. Our model is not a model for diagnosis or prediction of individual-level outcomes, and we do not have the relevant data to validate predictions.

**References**

1.	Johnson LF, Kubjane M, de Voux A, Ohrnberger J, Tlali M. An agent-based model of binge drinking, inequitable gender norms and their contribution to HIV transmission, with application to South Africa. BMC Infect Dis. 2023;23(1):500. doi: 10.1186/s12879-023-08470-y. PubMed Central PMCID: PMCPMC10385913.

2.	Johnson LF, Kubjane M, Moolla H. MicroCOSM: a model of social and structural drivers of HIV and interventions to reduce HIV incidence in high-risk populations in South Africa. BioRxiv. 2018:310763 [Preprint]. doi: 10.1101/310763

3.	Baird SJ, Garfein RS, McIntosh CT, Özler B. Effect of a cash transfer programme for schooling on prevalence of HIV and herpes simplex type 2 in Malawi: a cluster randomised trial. Lancet. 2012;379(9823):1320-9. doi: 10.1016/S0140-6736(11)61709-1.

4.	McGrath N, Nyirenda M, Hosegood V, Newell ML. Age at first sex in rural South Africa. Sex Transm Infec. 2009;85 (Suppl 1):i49-55. doi: 10.1136/sti.2008.033324. PubMed Central PMCID: PMC2654105.

5.	Anderson KG, Beutel AM, Maughan-Brown B. HIV risk perceptions and first sexual intercourse among youth in Cape Town South Africa. Int Fam Plan Perspect. 2007;33(3):98-105. doi: 10.1363/ifpp.33.098.07.

6.	Dunkle KL, Jewkes RK, Brown HC, Gray GE, McIntryre JA, Harlow SD. Transactional sex among women in Soweto, South Africa: prevalence, risk factors and association with HIV infection. Soc Sci Med. 2004;59(8):1581-92.

7.	Pitpitan EV, Kalichman SC, Eaton LA, Watt MH, Sikkema KJ, Skinner D, et al. Men (and women) as "sellers" of sex in alcohol-serving venues in Cape Town, South Africa. Prev Sci. 2014;15(3):296-308. doi: 10.1007/s11121-013-0381-y. PubMed Central PMCID: PMC3743930.

8.	Duby Z, Jonas K, McClinton Appollis T, Maruping K, Vanleeuw L, Kuo C, Mathews C. From survival to glamour: Motivations for engaging in transactional sex and relationships among adolescent girls and young women in South Africa. AIDS Behav. 2021;25(10):3238-54. doi: 10.1007/s10461-021-03291-z.

9.	Department of Health, Statistics South Africa, South African Medical Research Council, ICF. South Africa Demographic and Health Survey 2016. Pretoria: 2019. Available from: <https://www.dhsprogram.com/pubs/pdf/FR337/FR337.pdf>. Accessed 19 March 2019.

10.	Dunkle KL, Jewkes R, Nduna M, Jama N, Levin J, Sikweyiya Y, Koss MP. Transactional sex with casual and main partners among young South African men in the rural Eastern Cape: prevalence, predictors, and associations with gender-based violence. Soc Sci Med. 2007;65(6):1235-48.

11.	Jewkes R, Morrell R, Sikweyiya Y, Dunkle K, Penn-Kekana L. Men, prostitution and the provider role: understanding the intersections of economic exchange, sex, crime and violence in South Africa. PLoS One. 2012;7(7):e40821. doi: 10.1371/journal.pone.0040821. PubMed Central PMCID: PMCPMC3401205.

12.	Seidu AA, Darteh EKM, Kumi-Kyereme A, Dickson KS, Ahinkorah BO. Paid sex among men in sub-Saharan Africa: Analysis of the demographic and health survey. SSM Popul Health. 2020;11:100459. doi: 10.1016/j.ssmph.2019.100459. PubMed Central PMCID: PMCPMC7451820.

13.	Phiri M, Lemba M, Chomba C, Kanyamuna V. Examining differentials in HIV transmission risk behaviour and its associated factors among men in Southern African countries. Humanities and Social Sciences Communications. 2022;9(1):295. doi: 10.1057/s41599-022-01312-3. PubMed Central PMCID: PMCPMC9419142.

14.	Okafor CN, Christodoulou J, Bantjes J, Qondela T, Stewart J, Shoptaw S, et al. Understanding HIV risk behaviors among young men in South Africa: a syndemic approach. AIDS Behav. 2018;22(12):3962-70. doi: 10.1007/s10461-018-2227-x. PubMed Central PMCID: PMCPMC6330132.

15.	Lesotho Ministry of Health. Lesotho Population-based HIV Impact Assessment 2020 (LePHIA 2020): Final Report. Maseru: 2022.

16.	Wilson SJ, Tanner-Smith EE, Lipsey MW, Steinka-Fry K, Morrison J. Dropout prevention and intervention programs: effects on school completion and dropout among school-aged children and youth. Campbell Syst Rev. 2011:8. doi: 10.4073/csr.2011.8.

17.	Garcia S, Saavedra J. Educational impacts and cost-effectiveness of conditional cash transfer programs in developing countries: a meta-analysis. National Bureau of Economic Research, 2017 Working Paper 23594. Available from: <https://www.nber.org/papers/w23594>. Accessed 30 April 2023.

18.	Tripney JS, Hombrados JG. Technical and vocational education and training (TVET) for young people in low- and middle-income countries: a systematic review and meta-analysis. Empir Res Vocat Educ Train. 2013;5:3.

19.	Cho H, Mbai I, Luseno WK, Hobbs M, Halpern C, Hallfors DD. School support as structural HIV prevention for adolescent orphans in Western Kenya. J Adolesc Health. 2018;62(1):44-51. doi: 10.1016/j.jadohealth.2017.07.015. PubMed Central PMCID: PMCPMC5742035.

20.	Smith AFM, Gelfand AE. Bayesian statistics without tears - a sampling resampling perspective. Am Stat. 1992;46(2):84-8. doi: 10.2307/2684170.

21.	Neethling I, Lambert EV, Cois A, Roomaney RA, Awotiwon OF, Pacella R, et al. Estimating the changing burden of disease attributable to low levels of physical activity in South Africa for 2000, 2006 and 2012. S Afr Med J. 2022;112(8b):639-48. doi: 10.7196/SAMJ.2022.v112i8b.1648.

22.	GBD 2021 Risk Factors Collaborators. Global burden and strength of evidence for 88 risk factors in 204 countries and 811 subnational locations, 1990–2021: a systematic analysis for the Global Burden of Disease Study 2021. Lancet. 2024;403:2162-203.

23.	Herbst K, Juvekar S, Jasseh M, Berhane Y, Chuc NTK, Seeley J, et al. Health and demographic surveillance systems in low- and middle-income countries: history, state of the art and future prospects. Glob Health Action. 2021;14 (Suppl 1):1974676. doi: 10.1080/16549716.2021.1974676. PubMed Central PMCID: PMCPMC8986235.

24.	Allorant A, Kuchukhidze S, Stannah J, Xia Y, Masuku SS, Ekanmian GK, et al. Socio-demographic and geographic disparities in HIV prevalence, HIV testing and treatment coverage: An analysis of 108 national household surveys in 33 African countries. J Int AIDS Soc. 2025;28(8):e70024. doi: 10.1002/jia2.70024. PubMed Central PMCID: PMCPMC12350184.

25.	Karvonen S, Moisio P, Vepsalainen K, Ollonqvist J. Assessing health gradient with different equivalence scales for household income - A sensitivity analysis. SSM Popul Health. 2021;15:100892. doi: 10.1016/j.ssmph.2021.100892. PubMed Central PMCID: PMCPMC8368020.

26.	Yamanis TJ, Fisher JC, Moody JW, Kajula LJ. Young men's social network characteristics and associations with sexual partnership concurrency in Tanzania. AIDS Behav. 2016;20(6):1244-55. doi: 10.1007/s10461-015-1152-5. PubMed Central PMCID: PMCPMC4753135.

27.	Louw M, van der Berg S, Yu D. Convergence of a kind: educational attainment and intergenerational social mobility in South Africa. S Afr J Econ. 2007;75(3):548-71.

28.	Boyd J, Wilson R, Elsenbroich C, Heppenstall A, Meier P. Agent-based modelling of health inequalities following the complexity turn in public health: a systematic review. International Journal of Environmental Research and Public Health. 2022;19(24):16807. doi: 10.3390/ijerph192416807. PubMed Central PMCID: PMCPMC9779847.

29.	Stannah J, Flores Anato JL, Pickles M, Larmarange J, Mitchell KM, Artenie A, et al. From conceptualising to modelling structural determinants and interventions in HIV transmission dynamics models: a scoping review and methodological framework for evidence-based analyses. BMC Med. 2024;22:404.

30.	Hogan JW, Galai N, Davis WW. Modeling the impact of social determinants of health on HIV. AIDS Behav. 2021;25 (Suppl 2):215-24. doi: 10.1007/s10461-021-03399-2. PubMed Central PMCID: PMCPMC8414021.

31.	Swann M. Economic strengthening for HIV prevention and risk reduction: a review of the evidence. AIDS Care. 2018;30 (Suppl 3):37-84. doi: 10.1080/09540121.2018.1479029.

32.	Jennings L, Ssewamala FM, Nabunya P. Effect of savings-led economic empowerment on HIV preventive practices among orphaned adolescents in rural Uganda: results from the Suubi-Maka randomized experiment. AIDS Care. 2016;28(3):273-82. doi: 10.1080/09540121.2015.1109585. PubMed Central PMCID: PMCPMC4747687.

33.	Björkman Nyqvist M, Corno L, de Walque D, Svensson J. Using lotteries to incentivize safer sexual behavior: evidence from a randomized controlled trial on HIV prevention. World Bank Development Research Group, 2015. Available from: <https://openknowledge.worldbank.org/bitstream/handle/10986/21654/WPS7215.pdf?sequence=1>. Accessed 5 Nov 2015.

34.	Rubio FA, Amad AAS, Aransiola TJ, de Oliveira RB, Naidoo M, Moya EMD, et al. Evaluating social protection mitigation effects on HIV/AIDS and tuberculosis through a mathematical modelling study. Sci Rep. 2024;14(1):11739. doi: 10.1038/s41598-024-62007-0. PubMed Central PMCID: PMCPMC11111786.

35.	Bruniera de Oliveira RB, Rubio FA, Anderle R, Sanchez M, de Souza LE, Macinko J, et al. Incorporating social determinants of health into the mathematical modeling of HIV/AIDS. Sci Rep. 2022;12(1):20541. doi: 10.1038/s41598-022-24459-0. PubMed Central PMCID: PMCPMC9707122.

36.	Meyer-Rath G, Jamieson L, Mudimu E, Imai-Eaton JW, Johnson LF. The cost of the plunge: the impact and cost of a cessation of PEPFAR-supported services in South Africa. AIDS. 2025;39:1476-80. doi: 10.1101/2025.04.22.25326207.

37.	Remme M, Vassall A, Lutz B, Luna J, Watts C. Financing structural interventions: going beyond HIV-only value for money assessments. AIDS. 2014;28(3):425-34. doi: 10.1097/QAD.0000000000000076.

38.	Kilburn K, Thirumurthy H, Halpern CT, Pettifor A, Handa S. Effects of a large-scale unconditional cash transfer program on mental health outcomes of young people in Kenya. J Adolesc Health. 2016;58(2):223-9. doi: 10.1016/j.jadohealth.2015.09.023. PubMed Central PMCID: PMCPMC4724529.

39.	Baird S, De Hoop J, Ozler B. Income shocks and adolescent mental health. J Hum Resour. 2013;48(2):370-403.

40.	Haushofer J, Shapiro J. The short-term impact of unconditional cash transfers to the poor: experimental evidence from Kenya. Q J Econ. 2016;131(4):1973-2042. doi: 10.1093/qje/qjw025. PubMed Central PMCID: PMCPMC7575201.

41.	Carter DJ, Daniel R, Torrens AW, M NS, Maciel ELN, Bartholomay P, et al. The impact of a cash transfer programme on tuberculosis treatment success rate: a quasi-experimental study in Brazil. BMJ Glob Health. 2019;4(1):e001029. doi: 10.1136/bmjgh-2018-001029. PubMed Central PMCID: PMCPMC6347926.

42.	Ngamasana EL, Moxie J. Cash transfer, maternal and child health outcomes: a scoping review in sub-Saharan Africa. Glob Health Action. 2024;17(1):2309726. doi: 10.1080/16549716.2024.2309726. PubMed Central PMCID: PMCPMC10860414.

43.	Keyes CL. Social well-being. Soc Psychol Q. 1998;61(2):121-40.

44.	World Bank. World Development Indicators 2025 [1 Sept 2025]. Available from: <https://datatopics.worldbank.org/world-development-indicators/>.

45.	World Bank. Inequality in Southern Africa: an Assessment of the Southern African Customs Union. Washington DC, USA: 2022. Available from: <https://documents1.worldbank.org/curated/en/099125303072236903/pdf/P1649270c02a1f06b0a3ae02e57eadd7a82.pdf>. Accessed 14 Aug 2024.

46.	Johnson LF, Kubjane M, Ohrnberger J, Tlali M, editors. The contribution of binge drinking and inequitable gender norms to HIV transmission in South Africa [Abstract EPC153]. 24th International AIDS Conference; 2022 29 July-2 Aug; Montreal, Canada.

47.	Johnson LF, Geffen N. A comparison of two mathematical modeling frameworks for evaluating sexually transmitted infection epidemiology. Sex Transm Dis. 2016;43(3):139-46. doi: 10.1097/OLQ.0000000000000412.
