# Supplementary material for: Evaluation of economic strengthening in South Africa and its impact on HIV, sexually transmitted infections, and teenage births: A modelling study
Source: PLoS Med. 2025 Dec 18;22(12):e1004826. doi: 10.1371/journal.pmed.1004826 (PMC12714221; doi:10.1371/journal.pmed.1004826)
Supplement: Attachment — Submitted filename: Resubmission comments2.docx [file pmed.1004826.s005.docx]

Dear Dr Farrell

Thank you the positive feedback on our revised paper. We are grateful to you and the reviewers for the additional comments and suggestions, which have helped to further improve the paper. We have revised the paper based on the feedback received, and have responded below to the individual comments. Although none of the results in the main text have changed, we have added some additional results in the supplementary text, based on the feedback received. Please let us know if there are any further concerns.

Yours sincerely

Leigh Johnson

Comments from the academic editor:

* Please confirm that your title complies with PLOS Medicine's style. Your title must be nondeclarative and not a question. It should begin with main concept if possible. "Effect of" should be used only if causality can be inferred, i.e., for an RCT. Please place the study design ("A randomized controlled trial," "A retrospective study," "A modelling study," etc.) in the subtitle (ie, after a colon). Please consider: Evaluation of economic strengthening in South Africa and its impact on HIV, sexually transmitted infections and teenage births: a modeling study

**RESPONSE**: We have made the suggested change to the title.

* Please confirm that your abstract complies with our requirements, including format (three sections: Background, Methods and Findings, and Conclusions) and providing all the information relevant to this study type https://journals.plos.org/plosmedicine/s/submission-guidelines#loc-abstract

**RESPONSE**: We have changed the final sentence of the Background section of the abstract to “We aimed to model the effect of socio-economic status, and associated economic strengthening interventions, in South Africa.” We have also added the following sentence about limitations: “Key limitations include the assumption of uniform STI treatment access across socio-economic strata, and the exclusion of possible socio-economic effects at a community level.”

* Please ensure that the Introduction ends with a clear description of the study question or hypothesis.

**RESPONSE**: We have modified both the final sentence in the Background section of the abstract (as noted previously), and have also modified the final sentence in the Introduction: “Building upon recent advances in the use of agent-based models to represent socio-economic differences in health outcomes [1], we aim to assess which economic strengthening interventions are likely to have the greatest impact on HIV, STIs and teenage births.”

* Please ensure that all abbreviations are defined at first use throughout the text.

**RESPONSE**: We have added the definition of odds ratio (OR) at first mention. The only other acronyms we have used are APCHI, ART, CI, COVID, HIV, PAF, PRCC, RCT, STI and VMMC. With the exception of COVID and HIV (which are widely known), all have been defined at first use. Some additional acronyms are used in the tables, but these are defined in the table legends.

* Please confirm that all numbers presented in the abstract are present and identical to numbers presented in the main manuscript text.

**RESPONSE**: We have double-checked that the numbers in the abstract are identical to those in the main text.

Line 49 of Background: Qualify ‘these’. Incidences?events?

**RESPONSE**: We have clarified “these incidence rates”.

Line 53: The first sentence should explain what you did in this study. Please rephrase to indicate what the model was used to assess (reword “to reflect effects of”).

**RESPONSE**: We have changed “to reflect effects of” to “to assess effects of”.

Line 54: remove comma after Africa

**RESPONSE**: We have removed the comma.

Please use active voice throughout. E.g. “We estimated…”

**RESPONSE**: We have changed to active voice throughout the text, except in a handful of cases where the use of ‘we’ would have been overly repetitive.

Please use commas rather than hyphens in confidence intervals.

**RESPONSE**: We have replaced the hyphens with commas in all confidence intervals. This has made the text in the abstract a bit cumbersome, and we have tried to rephrase to maintain clarity. We would welcome alternative suggestions.

Lines 65-66, impact used twice. Please clarify what the first ‘impact’ relates to (which outcomes).

**RESPONSE**: We have changed the sentence to “Over 2025-2040, none of the interventions are estimated to *reduce HIV, STIs or teenage births significantly*, due to limited impact on secondary economic outcomes.”

Line 66 and lines 109-110: please rephrase “would come close”. Please be precise in how you frame the reduction in teenage births. Please also do not repeat phrasing of the Abstract in the Author Summary.

**RESPONSE**: We have removed this sentence from the author summary, as it is not a key result. In the abstract we have modified the sentence as follows: “The greatest impact would be that of school support on teenage births (a 5% reduction, 95% CI: -1,12%).”

* In the last sentence of the Abstract Methods and Findings section, please describe the main limitation(s) of the study's methodology.

**RESPONSE**: We have added the following sentence: “Key limitations include the assumption of uniform STI treatment access across socio-economic strata, and the exclusion of possible socio-economic effects at a community level.”

Please add an additional qualifier to the last sentence of the Abstract to explain what the recently trialled econonomic interventions aimed to do.

**RESPONSE**: The phrase “economic strengthening interventions” was previously explained in the abstract (we specifically mentioned the three types of interventions that were considered). We do not think it is necessary to repeat the explanation in the concluding sentence.

In the final bullet point of ‘What Do These Findings Mean?’, please include the main limitations of the study in non-technical language.

**RESPONSE**: We have added the following sentence in the final bullet: “A limitation is that some socio-economic effects were not represented in the model, e.g. effects of socio-economic status on access to STI treatment, and effects of community socio-economic status.”

In the main text, Background should be called Introduction.

**RESPONSE**: We have made this change.

Line 191: qualify ‘impactful’ (what do they impact?).

**RESPONSE**: We have changed “to be most impactful” to “to have the greatest impact on HIV, STIs and teenage births”.

Please provide a 1-2 sentence introduction to the Results section.

**RESPONSE**: We have added the following sentence: “We present the results in two parts, the first describing the results of the model calibration and the role of socio-economic status in the period up to 2020, and the second describing possible future changes if economic strengthening interventions were to be introduced.”

Please clarify line 468 for the general reader.

**RESPONSE**: Apologies, there was a missing word here, and the word “mediate” was perhaps misleading/unclear. We have changed the text in brackets to “the latter two effects being *due to* *assumed* effects of education *on* inequitable gender norms, and *assumed* effects of inequitable gender norms on male risk behaviour [2]”. We assume it was not the term “inequitable gender norms” that was the source of confusion.

The figure legend to Figure 2 should not start with definitions of acronyms. Please revise start with an explanation of what is in the figure. Each figure panel should be described separately.

**RESPONSE**: We have moved the acronyms to the end of the legend. We have also added the following sentences at the start of the legend: “Model estimates of key reproductive health outcomes and economic indicators are compared against calibration targets. Reproductive health outcomes include HIV prevalence (panel a) and HIV incidence (panel b), both in 15-49 year olds, STI incidence per 100 000 population aged 15-49 (panel c), and total births to teenage mothers (panel d). Economic indicators include the proportion of youth who have completed high school (panel e), the proportion of 15-64 year olds who are employed (panel f), median monthly household income per household member (panel g) and Gini coefficient (panel h).”

* In the abstract, please include the important dependent variables that are adjusted for in the analyses.

**RESPONSE**: We are not using a regression modelling approach, and as such there is no ‘adjustment’.

* Where data points are discrete, please ensure that they are depicted in the figures as discrete data and not as a continuous line.

**RESPONSE**: In Figure 2, the data are all presented as discrete points (dots) and not as continuous lines.

* Please provide the unadjusted comparisons as well as the adjusted comparisons in all relevant Tables.

**RESPONSE**: As noted before, we are not using a regression modelling approach, and as such there is no ‘adjustment’. However, it is perhaps worth noting that the partial rank correlation coefficients (PRCCs) in Table 3 are calculated based on a regression model. The equivalent ‘unadjusted’ correlation coefficients are shown in the table below, and are compared against the partial rank correlation coefficients that were previously reported. We see that several of the relationships that are significant in the multivariable analysis (PRCCs) are not significant in the unadjusted analysis (when calculating standard correlation coefficients). This is to be expected, as the effect of some parameters can only be seen clearly when controlling for other parameters that explain more of the variation in the PAFs. It is also partly explained by the fact that rank correlation measures are more sensitive to non-linear relationships than regular correlation coefficients. Only one relationship that is significant in the unadjusted analysis (the relationship between the HIV PAF and the effect of male employment on entry into casual sex) is non-significant in the multivariable analysis, which we interpret as a ‘type I error’ (since the correlation coefficient of 0.25 is close to the significance threshold of 0.22, and since it is counter-intuitive that a greater effect of male employment on entry into casual sex would *increase* the PAF for poverty). Since it is conventional in uncertainty analysis to report PRCCs rather than correlation coefficients, we have placed this table in the supplementary materials (Table S33), and referred to it in the main text.

Table S33: Correlates of population attributable fractions

| Parameter | Correlation coefficients | | | PRCCs | | |
| --- | --- | --- | --- | --- | --- | --- |
|  | HIV | STIs | Teenage  births | HIV | STIs | Teenage  births |
| OR for consistent condom use per year of schooling | **0.88** | **0.86** | 0.00 | **0.87** | **0.90** | **0.57** |
| Increase in casual sex in men who are employed | **0.25** | 0.16 | 0.02 | -0.09 | -0.05 | -0.01 |
| RR of commercial sex in men who are employed | 0.06 | -0.01 | 0.16 | 0.04 | -0.16 | -0.11 |
| Increase in casual sex in females, per log reduction  in APCHI^*^ | -0.11 | -0.05 | -0.09 | -0.14 | -0.04 | -0.12 |
| RR of partner acquisition in men who are employed | 0.09 | 0.07 | 0.10 | **0.30** | **0.27** | **0.27** |
| Increase in sexual debut in females per log reduction  in APCHI^*^ | 0.20 | 0.18 | 0.19 | 0.13 | 0.15 | 0.07 |
| RR of medical male circumcision per log increase  in APCHI | 0.08 | 0.11 | 0.06 | -0.08 | -0.09 | 0.01 |
| RR of sexual debut in females if currently in school | -0.18 | **-0.27** | **-0.77** | **-0.63** | **-0.87** | **-0.95** |
| RR of sexual debut in males if currently in school | -0.08 | -0.14 | -0.03 | -0.05 | -0.01 | **-0.27** |

Values show the correlation between the input parameters (Table 1) and the population attributable fractions (Table 2); values in bold indicate correlation coefficients that are statistically significant at the 5% level. * Per unit difference between the natural log of the APCHI and the national average log APCHI, for households that have a log APCHI below the national average (for those above the average, no income effect is modelled).

APCHI = adjusted per capita household income. OR = odds ratio. PRCC = partial rank correlation coefficient. RR = relative rate.

Modeling studies should include the following components, as derived from Geoffrey P Garnett, Simon Cousens, Timothy B Hallett, Richard Steketee, Neff Walker. Mathematical models in the evaluation of health programmes. (2011) Lancet DOI:10.1016/S0140-6736(10)61505-X:

**RESPONSE**: We responded to these points in our previous resubmission, but have copied and pasted the responses again, for completeness.

* Please provide a diagram that shows the model structure, including how the disease natural history is represented, the process and determinants of disease acquisition, and how the putative intervention could affect the system.

**RESPONSE**: This is included in Figure 1.

* Please provide a complete list of model parameters, including clear and precise descriptions of the meaning of each parameter, together with the values or ranges for each, with justification or the primary source cited, and important caveats about the use of these values noted.

**RESPONSE**: We have done this in Table 1. It is perhaps worth noting that there are many hundreds of parameters in the model, and it is not feasible to detail all of them, but Table 1 focuses on the parameters most relevant to the current study. Other parameters are explained in the supplementary materials or in previous published description of the model, which we have cited throughout our submission [2-4].

* Please provide a clear statement about how the model was fitted to the data including where relevant goodness-of-fit measure, the numerical algorithm used, which parameter varied, constraints imposed on parameter values, and starting conditions.

**RESPONSE**: A brief summary of these aspects is provided in the main text: the ‘Model calibration’ section in the Methods summarizes the Bayesian calibration approach and the likelihood calculation (based on the log difference between the modelled and observed trial outcomes), and Table 1 shows the parameters that were varied in the calibration process. In the supplementary materials we provide more detail: section 2.2 describes the likelihood calculation, section 2.3 describes the numerical algorithm (Sampling Importance Resampling) and the constraints imposed on parameters are shown in section 2.1 (in the case of gamma priors, any positive value is permissible, and in the case of beta priors, any value between 0 and 1 is permissible). The starting conditions for the socio-economic parameters are described in sections 1.1.3 and 1.2.2 of the supplementary materials.

* For uncertainty analyses, please state the sources of uncertainties quantified and not quantified this can include parameter, data, and model structure.

**RESPONSE**: We have evaluated parameter uncertainty, and the specific parameters for which we consider uncertainty are shown in Table 1 (as indicated in the footnote, wherever a standard deviation (SD) is specified, this represents a parameter that is varied in the model calibration and uncertainty analysis). We do not consider uncertainty regarding the model structure; structural uncertainty would normally be represented by the comparison of different models, but given that this is a novel area, there are no other models that we can compare against. Uncertainty in the data used in calibration (the randomized controlled trial data) is not evaluated explicitly, but by using a random effect model in the specification of the likelihood, we are implicitly allowing for uncertainty regarding the applicability of the data in the South African setting.

* Please provide sensitivity analyses to identify which parameter values are most important in the model. Uncertainty estimates seek to derive a range of credible results on the basis of an exploration of the range of reasonable parameter values. The choice of method should be presented and justified.

**RESPONSE**: In Table 3 we use partial rank correlation coefficients (PRCCs) to show which parameters are most critical to the variation in population attributable fractions, and in the last paragraph of the Results we present similar PRCCs for the intervention impacts. We have also used Bayesian calibration techniques, which are appropriate to determining uncertainty ranges around all model outputs. Both aspects are described in the Methods.

* Please discuss the scientific rationale for this choice of model structure and identify points where this choice could influence conclusions drawn. Please also describe the strength of the scientific basis underlying the key model assumptions.

**RESPONSE**: We previously amended the Background section to clarify the rationale for using a modelling approach: “They [models] also play a role in translating evidence from RCTs, which produce short-term estimates of impact at an individual or community level, into estimates of likely longer-term impact at a population level*.*” We also previously added a sentence to explain the rationale for an agent-based model specifically: “Agent-based models, which represent individual-level variation in health risk exposure and outcomes, are well suited to representing complex causal pathways and diverse outcomes, and are increasingly utilized in assessing socio-economic disparities in health [1]*.*” In the Discussion, we noted the limitation that we have not modelled the influence of income inequality at a community level, and this may have led to us understating the impacts of economic strengthening. We have been rigorous in setting the prior distributions in such a way that they reflect the uncertainty around how much of the observed associations between socioeconomic status and sexual behaviour are truly causal relationships – this is described in section 2.1 of the supplementary materials. In the Discussion, we have also stressed the uncertainty regarding the socio-economic effects on behavioural parameters, and the resulting wide confidence intervals around the model estimates.

Comments from Reviewers:

Reviewer #1: I appreciate the authors' thoughtful and constructive revisions, which have notably improved the transparency and methodological rigour of the manuscript. The majority of my previous concerns—pertaining to the model structure, calibration methodology, justification of assumptions, and the realism of intervention scenarios—have been adequately addressed. The revised version presents clearer descriptions of the model submodules, a more detailed explanation of the calibration procedures and prior specification, and an expanded discussion on the limitations related to generalisability and implementation feasibility.

While the manuscript is now suitable for publication, I offer the following final suggestions that may enhance its robustness and credibility:

1. Consider reporting basic model fit metrics (e.g., root mean square error, mean absolute error) or presenting posterior predictive checks for key outcomes to more clearly demonstrate the model's internal and external validity beyond qualitative assessments.

**RESPONSE**: We have added Table S31 in section 2.5 of the supplementary materials showing the root mean square error and mean absolute error for each of the intervention types. The root mean square error and mean absolute error statistics were calculated using the 100 parameter combinations in the posterior sample, and are calculated on a log scale. The model fits to the RCT data are best in the case of vocational training interventions, followed by cash transfer interventions.

Table S31: Model fit statistics

|  | Cash transfer | School support | Vocational training | Total |
| --- | --- | --- | --- | --- |
| Root mean square error | 0.521 | 0.645 | 0.333 | 0.537 |
| Mean absolute error | 0.287 | 0.432 | 0.267 | 0.327 |

2. Given the wide credible intervals observed for several outcomes, it would be helpful to provide a more explicit narrative interpretation of these intervals and their implications for uncertainty in policy recommendations.

**RESPONSE:** We have already acknowledged this limitation at a number of points. But since the reviewer specifically refers here to “implications for uncertainty in policy recommendations”, it is perhaps worth noting that the confidence intervals are widest in the case of the PAF estimates, which are of less direct policy relevance. The confidence intervals are much narrower in the case of the estimated future intervention impacts. For example, the effects of the cash transfer intervention on HIV incidence and STI incidence, over the 2025-2040 period, have confidence intervals of (-4,4%) and (-1,1%) respectively, and in response to point g of the reviewer’s previous comments, we stressed that these are the most extreme effects we are likely to see, under conditions of perfect implementation of the interventions. In other words, the range of uncertainty is likely to be narrower than these ranges suggest (closer to zero) under ‘real world’ conditions. As such, we do not feel we are overstating when we conclude that “Recently trialled economic strengthening interventions have insufficient impact on socio-economic status to reduce HIV and STIs significantly at a population level.” We are willing to consider proposed wording changes, but it is not currently clear to us where the policy implications are overstated.

3. While parameter uncertainty is well-addressed, the manuscript would benefit from additional sensitivity analysis—or at least discussion—regarding structural assumptions, particularly the simplified causal pathways linking socioeconomic status (SES) to health-related behaviours.

**RESPONSE**: In the opening pages of section 1.4 of the supplementary materials, we discussed some of the structural uncertainties, particularly regarding the choice of socio-economic effects and the complexities around relative versus absolute measures of deprivation. We have moved some of this into the Discussion section of the main paper: “Different measures of socio-economic status can influence risk behaviour in different ways, but in the interests of model parsimony we have limited our model to one socio-economic effect on each risk behaviour. There is also uncertainty regarding the relative importance of absolute and relative measures of deprivation in driving risk behaviour [5, 6]; in our model we assume a combination of both. In line with ‘Diffusion of innovations’ theory [7] and the ‘Inverse equity hypothesis’ [8], we have assumed increases in condom use in the early stages of the HIV epidemic follow a strong socio-economic gradient, but that this gradient reduces over time; further work is required to assess the validity of this assumption, and whether it applies to other HIV interventions. These complexities are discussed in further detail in section 1.4 of S1 Text.” A number of other sources of structural uncertainty have already been discussed (e.g. not modelling intergenerational transfers, social network effects, or the effects of socio-economic status at a community level).

4. The model currently assumes immediate behavioural effects following changes in SES. Future iterations could improve realism by incorporating lagged or cumulative effects, especially for longer-term interventions such as educational and vocational support.

**RESPONSE**: The model does already include several lagged effects. Educational interventions, as we model them, have both an immediate and a lagged effect. The immediate effect is due to both (a) the increase in school retention (and the associated reduction in rates of sexual debut) and (b) the material component of the school support (effectively increasing household income). The lagged effect is due to the increase in educational attainment, which in the longer term is assumed to increase levels of condom use, marriage, hormonal contraceptive use and HIV testing. The vocational training interventions, as we model them, also have a combination of short-term and long-term effects. The short-term effect is due to higher probability of employment, but this effect is compounded in the longer term because previous employment is assumed to increase the odds of later employment. However, cash transfer interventions are modelled as having only short-term effects (because they increase household income, they reduce women’s short-term incentive to engage in transactional sex and early sexual debut). The only long-term effect that we are aware of, which is not represented in the model, is the effect of intergenerational transfers in education (this limitation is already acknowledged in the third-last paragraph of the discussion).

Reviewer #2: Most of the comments from the previous reviewers were well addressed, but I still have the remaining concerns:

* Behavioral Mechanism Precision (Reviewer #1, point b): The authors provide detailed explanations of behavioral mechanisms in the supplementary materials and seem hesitant to duplicate this information in the main text due to length concerns. The reviewer may still feel this area could benefit from more inclusion in the main body of the paper.

**RESPONSE**: Reviewer 1 has commented on our revised paper, and did not request that we move the material into the main text. However, as noted in response to point 3 by Reviewer 1 (above), we have added further text to describe uncertainties around some key behavioural assumptions: “There is also uncertainty regarding the relative importance of absolute and relative measures of deprivation in driving risk behaviour [5, 6]; in our model we assume a combination of both. In line with ‘Diffusion of innovations’ theory [7] and the ‘Inverse equity hypothesis’ [8], we have assumed increases in condom use in the early stages of the HIV epidemic follow a strong socio-economic gradient, but that this gradient reduces over time; further work is required to assess the validity of this assumption, and whether it applies to other HIV interventions.”

* Implementation of Interventions (Reviewer #1, point g): The authors acknowledge that the assumptions about intervention implementation are optimistic but justify it as a "best-case scenario" assessment. The reviewer might still prefer a more realistic scaling scenario, even if briefly explored.

**RESPONSE**: Reviewer 1 has commented on our revised paper, and did not ask that we include more realistic scenarios. As we noted in our previous response to reviewer 1, the scenarios were intentionally optimistic, and we responded to Reviewer 1 by acknowledging this in the Discussion, noting that this reinforced the point that the interventions are unlikely to have an impact in a more plausible “real world” setting. Using more conservative assumptions about intervention uptake/implementation would only shrink the means and confidence intervals in Table 4 towards zero (and they are already very close to zero in most cases), which would not qualitatively change our conclusions.

* Model structure and scientific rationale (Reviewer #2, point 3): The authors added a few sentences in the Introduction to make the rationale clearer. "They [mathematical models] also play an important role in translating evidence from randomized trials, which produce short-term estimates of impact at an individual or community level, into estimates of likely longer-term impact at a population level.... Agent-based models, which represent individual-level variation in health risk exposure and outcomes, are well suited to representing complex causal pathways and diverse outcomes, and are increasingly utilized in assessing socio-economic disparities in health [28]." This response only addresses why modeling is important, but did not mention the rationale for the model structure.

**RESPONSE**: We have added the following sentences in the paragraph following Figure 1: “Socio-economic status has a number of dimensions, which can influence sexual behaviour in different ways, and socio-economic status is often measured in terms of household wealth/income [9]. We therefore chose to model socio-economic status based on a combination of household- and individual-level characteristics.”

* The reasons for this lack of impact should be explored in more detail (Reviewer #2, point 14): The authors showed in Figure 3 that the interventions have only a modest impact on socioeconomic indicators, and we have added further detail to Table 4 showing that the interventions have almost zero impact on intermediate behavioural outcomes. "These modest effects on socioeconomic indicators are further diluted at each subsequent step in the causal pathway, so that the final effects on reproductive health outcomes are negligible." However, the reasons for this lack of impact is still not clear.

**RESPONSE**: There are several factors to consider, in addition to the ones mentioned in our previous response. Firstly, as we have noted in the first paragraph of the Discussion, there are a combination of positive and negative effects for most interventions, which partially offset one another (for example, a vocational training intervention may reduce risk behaviours through increased household incomes, but increase risk behaviours through increased male employment). Secondly, when we specified the prior distributions for the socio-economic effects on sexual risk behaviour, we attached non-zero probabilities to the possibility of no effect, and the parameter combinations with no effect contribute to the lack of effect (as well as the imprecision). Thirdly, the RCT data to which we are calibrating the model are too heterogeneous and too imprecise (in most instances) to pull the posterior means away from the prior means (hence there are relatively few cases of significant differences between the prior and posterior estimates in Table S30). This further contributes to the lack of precision. Fourthly, there is stochastic variation, as this is an agent-based model, and this further contributes to model imprecision. We have added the following sentences in the Discussion: “A number of other factors may explain the non-significant intervention impacts. Firstly, we used hurdle distributions to represent prior uncertainty in key parameters, which is conservative because a substantial probability is assigned to a null relationship between each socio-economic variable and sexual risk behaviour. Secondly, and closely related, the RCT data to which we are calibrating the model are too heterogeneous and too imprecise (in most instances) to pull the posterior means away from the prior means (hence there are relatively few cases of significant differences between the prior and posterior estimates in Table S30). Thirdly, the model is agent-based and therefore stochastic, and stochastic variation also contributes to the imprecision.”

* Number of Simulations (Reviewer #1, point f): The authors doubled the posterior sample size and the results have been updated. But authors acknowledge that this only partially addresses the reviewer's concern, they have mentioned constraints of computing power.

**RESPONSE**: Reviewer 1 was satisfied with our response. Although we acknowledged the limitation, it is important to note that the key results and conclusions did not change materially between the first submission and the re-submission, despite a new (and larger) sample being drawn from slightly different prior distributions. Although a larger sample size would have been desirable, it’s also important to note that this would not necessarily have solved the problem of wide confidence intervals. The wide confidence intervals are a reflection of real posterior uncertainty, and stochastic variability is only one contributing factor. The problem of imprecision is therefore deeper than just computing constraints.

* Accounting for Generalizability of Trial Populations (Reviewer #1, point j): The authors state that the focus is on the heterogeneity in effects across settings and they address this by including a random effect term in the likelihood function. However, this cannot address the problem of generalizability. Instead, the authors would test the difference between the trial populations and the generalized population, and generalize those through modeling using through g-formula or inverse probability weighting.

**RESPONSE**: We apologize that the response was confused. We cannot generalize from the trial population data. As we see it, there are two major challenges to generalizability, which perhaps require different responses. The first challenge, which the reviewer alludes to, is that within a particular population, the subset of people enrolled in an RCT is unlikely to be representative of the total population (or all the people who might be eligible to receive the intervention). We noted in the response to reviewer 1 that we simulated interventions only in individuals whose household income were below the national average, to achieve better alignment between the simulated and actual trial populations. Although we did not mention it in our previous response to reviewer 1, it is perhaps also worth noting that when we calibrated our model to RCT data, we matched the simulated trial population to the actual trial population based on age, sex, schooling and employment, again to avoid assuming that intervention effects are the same across age/sex/socio-economic strata (this point was made in section 2.2.4 of the supplementary materials: “The modelled eligible population is further restricted to match the age, sex and schooling/employment profile of the population recruited into the corresponding RCT.”). Techniques such as g-formula and inverse probability weighting are appropriate for improving generalizability within a population, but they require individual-level data, and our analysis is based only on aggregated RCT data. Assumptions about heterogeneity in intervention effects are already implicit in our model (for example, young adults may benefit more from vocational training because they experience greater barriers to entering the job market than older adults). While we cannot generalize from a trial sub-population to the general population, we are weighting the heterogeneous modelled intervention effects (across different sub-populations) by the size of each eligible sub-population, so that the weighted average is more representative of what we might expect in the general population, if the intervention were offered to everyone who is eligible.

The second challenge, which we were more concerned with when previously responding to reviewer 1, is that even if an RCT perfectly represents the population in a particular location, its findings might not be applicable in other districts/countries/regions, either because of the uniqueness of the population or the uniqueness of the intervention design. In the same way that one might reduce bias in a meta-analysis of RCTs by using random effects (rather than fixed effects), we are using random effects in the specification of the likelihood to avoid giving excessive weight to individual (typically larger) studies in our model calibration. Challenges in generalizability remain in meta-analyses even when random effects are used, and the same is true in our analysis. We have acknowledged the challenges in generalizability in the second-last paragraph: “Our results might not be generalizable to other African settings, where there is typically less income inequality but more absolute poverty. In a recent analysis of national household survey data, HIV prevalence was inversely associated with education in southern and East Africa, but not in West and Central Africa [10].”

**References**

1.	Boyd J, Wilson R, Elsenbroich C, Heppenstall A, Meier P. Agent-based modelling of health inequalities following the complexity turn in public health: a systematic review. International Journal of Environmental Research and Public Health. 2022;19(24):16807. doi: 10.3390/ijerph192416807. PubMed Central PMCID: PMCPMC9779847.

2.	Johnson LF, Kubjane M, de Voux A, Ohrnberger J, Tlali M. An agent-based model of binge drinking, inequitable gender norms and their contribution to HIV transmission, with application to South Africa. BMC Infect Dis. 2023;23(1):500. doi: 10.1186/s12879-023-08470-y. PubMed Central PMCID: PMCPMC10385913.

3.	Johnson LF, Kubjane M, Moolla H. MicroCOSM: a model of social and structural drivers of HIV and interventions to reduce HIV incidence in high-risk populations in South Africa. BioRxiv. 2018:310763 [Preprint]. doi: 10.1101/310763

4.	Johnson LF, Geffen N. A comparison of two mathematical modeling frameworks for evaluating sexually transmitted infection epidemiology. Sex Transm Dis. 2016;43(3):139-46. doi: 10.1097/OLQ.0000000000000412.

5.	Eibner C, Evans WN. Relative deprivation, poor health habits, and mortality. J Hum Resour. 2005;40(3):591-620.

6.	Mishra S, Novakowski D. Personal relative deprivation and risk: An examination of individual differences in personality, attitudes, and behavioral outcomes. Personality and Individual Differences. 2016;90:22-6.

7.	Dearing JW, Cox JG. Diffusion Of innovations theory, principles, and practice. Health Affairs. 2018;37(2):183-90. doi: 10.1377/hlthaff.2017.1104.

8.	Victora CG, Vaughan JP, Barros FC, Silva AC, Tomasi E. Explaining trends in inequities: evidence from Brazilian child health studies. Lancet. 2000;356:1093-8.

9.	Galobardes B, Shaw M, Lawlor DA, Lynch JW, Davey Smith G. Indicators of socioeconomic position (part 1). J Epidemiol Community Health. 2006;60(1):7-12. doi: 10.1136/jech.2004.023531. PubMed Central PMCID: PMCPMC2465546.

10.	Allorant A, Kuchukhidze S, Stannah J, Xia Y, Masuku SS, Ekanmian GK, et al. Socio-demographic and geographic disparities in HIV prevalence, HIV testing and treatment coverage: An analysis of 108 national household surveys in 33 African countries. J Int AIDS Soc. 2025;28(8):e70024. doi: 10.1002/jia2.70024. PubMed Central PMCID: PMCPMC12350184.
